# Investigation of an Autochthonous Outbreak of Bovine Besnoitiosis in Northwestern Sicily

**DOI:** 10.3390/pathogens11020122

**Published:** 2022-01-20

**Authors:** Veronica Cristina Neve, Miriana Coltraro, Alessandro Stamilla, Filippo Spadola, Roberto Puleio, Guido Ruggero Loria, Francesco Antoci, Giuseppe Cascone, Felice Salina

**Affiliations:** 1 Department of Veterinary Science, University of Messina, 98168 Messina, Italy; veronicacristina.neve@unime.it (V.C.N.); filippo.spadola@unime.it (F.S.); 2 Istituto Zooprofilattico Sperimentale of Sicily, 90129 Palermo, Italy; miriana.coltraro@gmail.com (M.C.); roberto.puleio@izssicilia.it (R.P.); guidoruggero.loria@izssicilia.it (G.R.L.); antocif@gmail.com (F.A.); giuseppe.cascone60@gmail.com (G.C.); felice.salina@izssicilia.it (F.S.); 3 Department of Agriculture, Food and Environmental Science (Di3A), University of Catania, 95124 Catania, Italy

**Keywords:** *Besnoitia besnoiti*, bovine besnoitiosis, cattle, histopathology, serology, Sicily, skin disease, protozoa

## Abstract

Bovine besnoitiosis is a parasitic disease caused by the protozoan *Besnoitia besnoiti*, leading to infertility in bulls and abortions in cows. In Italy, it is considered an emerging disease, recently introduced by the importation of animals from Spain and France. In the last decade, many outbreaks have been reported and confirmed in native cattle, mostly in northern and central Italy. This study reports on an autochthonous outbreak of bovine besnoitiosis in two nearby farms located in northwestern Sicily. A 15-month-old Limousine bull born on the farm showed typical clinical signs of the chronic disease phase, such as edema of the scrotum with subsequent hyperkeratosis associated with eschars and thickening of the skin. A histopathological examination revealed the presence of *Besnoitia* tissue cysts containing bradyzoites in the eyes, tendons, testicles, dermis, and nictitating membrane. A serological investigation using a commercial ELISA kit revealed a high seroprevalence of the antibody anti-*B. besnoiti* (79.2% for the farms in this study). Clinical disease showed low prevalence (1.5%) despite the high seroprevalence of specific antibodies in the herd, confirming that bovine besnoitiosis is an emergent endemic pathogen in Sicily, but its clinical behavior still remains sporadic.

## 1. Introduction

Bovine besnoitiosis is caused by an infection of the intracellular protist parasite *Besnoitia besnoiti*, belonging to the genus Besnoitia, family Sarcocystidae, and phylum Apicomplexa. To date, 10 different species that cause infection have been identified, but only *B. besnoiti* causes several kinds of cutaneous damage and infertility in cattle. The lifecycle of *B. besnoiti* is still unclear because the definitive host is still unknown [1]. Otherwise, it is well known that one of the intermediate hosts is the bovine. Transmission in cattle is thought to occur through ingestion of the sporulated oocysts in the feces of definitive hosts, by some bloodsucking insects that can directly transfer parasites from one animal to another or more frequently by natural mating. Infection due to insect bite seems to be more common than infection caused by oocyst ingestion, because the supposed final host could be a felid, as well as for the other species of Besnoitia [2].

The entire lifecycle of this protist parasite remains unknown, but it is suspected that *B. besnoiti* has two host (heteroxenous) lifecycles, with a final host still unknown, and different intermediate hosts such as cattle [1]. Upon infection, the tachyzoites, carried by insects or transmitted by natural mating [3], travel through blood vessels, reaching different parts of the body to proliferate in endothelial cells and fibroblasts [4]. The horizontal transmission of bradyzoites and tachyzoites between intermediate hosts has been shown experimentally [4,5].

Cattle seem to be an important intermediate host because they harbor tachyzoites and cyst-forming bradyzoites. Basically, in cattle it is possible to distinguish two asexual infectious stages: acute and chronic. During the acute infection phase, tachyzoites can rapidly replicate in the endothelium of blood vessels, where repeated host cell invasion and lysis can lead to the destruction of vascular endothelial cells [6]. In vitro studies have demonstrated that *Besnoitia* spp. proliferation is rather asynchronous, because different proliferation stages can be present at the same time; this might lead to an extended time period during which *Besnoitia* spp. invade their host cells [7]. In the chronic infection phase, bradyzoites are slowly replicated inside macroscopic cysts in the cells of the subcutaneous connective tissue, persisting in the host. Cyst formation begins about a week after the initial proliferation cycle [8], but it has not been demonstrated how long the cysts persist in cattle and if besnoitiosis can be reactivated from the chronic to the acute stage [9]. In cattle, it is possible to distinguish very early cysts measuring 15–25 µm 10 days after infection, which become 30–100 µm larger after 16–25 days. The organs most affected by cyst formation are the mucosae of the upper respiratory tract, the genitals, the scleral conjunctiva of the eyes, the dermis, and the tendons of the lower limbs [10]. The tissue cyst distribution is very distinctive in the skin and other organs. In the skin, cysts are more frequently observed in the papillary layer rather than the reticular one, while in highly vascularized tissues such as the brain, heart, spleen, kidneys, or liver, tissue cysts have rarely been observed [11]. Moreover, many tissue cysts are also found in nonintestinal mucosae, fasciae, and subcutaneous and intramuscular connective tissue [12]. The distribution pattern is still unknown, but it could be influenced by transmission and by the promotion of myofibroblasts during acute and subacute stages [6]. Besnoitiosis in cattle is considered a chronic and debilitating disease characterized by weight loss, a decrease in milk production, skin lesions, and transient or permanent sterility in breeding males. Consequently, it causes serious economic losses in livestock farms [3,13]. Although this disease is rarely fatal, the recovery period is often protracted; therefore, farmers generally prefer to cull infected animals [14]. In Europe, bovine besnoitiosis was described in 2010 as an emerging disease by the European Food Safety Authority (EFSA) [15], and in the last decade it has become an endemic disease in France, Spain, and Portugal [16]. Moreover, several cases of besnoitiosis have been confirmed in other European countries, including Germany, Switzerland, Hungary, Croatia, Belgium, and Ireland [13,17]. In Italy, although the majority of cases were related to imported stocks of animals from France [17], some endemic outbreaks have been confirmed in the northern regions, while a few cases have been confirmed in central and southern regions [18,19] and only two in Sicily [20,21].

Aside from the spread of the disease in the Italian peninsula, this study examines another endemic outbreak of bovine besnoitiosis in Sicily in 2020, which occurred in two farms located in the northeast of the region. The aim of this study is to contribute to the epidemiological knowledge of the clinical behavior and pathology of this protozoon infection, in order to prevent its diffusion and reduce the related economic losses. The prevalence of besnoitiosis in this area has been established by a serological, clinical, and histopathological survey.

## 2. Results

A total of 111 serum samples collected from two different farms of Limousine cattle were analyzed to test seroprevalence in the herd. The study was conducted after the discovery of clinical signs peculiar to bovine besnoitiosis. A total of 68 animals tested seropositive by ELISA: 11 on farm A and 57 on farm B (28.20% and 79.17% of the herds, respectively). Histopathological examination of tissue cysts confirmed infection by *B. besnoitiosis*.

### 2.1. Farm A

#### 2.1.1. Serological Findings

A total of 39 animals (30 female, 9 male) were screened by ELISA test in farm A. In total, 10 cows and 1 bull (28.20%) showed the presence of specific antibodies. Positive animals belonged to a highly variable age group ranging from 1 to 11 years old (Table 1).

#### 2.1.2. Clinical Signs

In April, two cows of nine years old showed some clinical signs including hyperthermia (41 °C), edema affecting muzzle and joints, cyanosis of the udder and the muzzle, and lacrimation and hyperemia of the conjunctiva, followed by folding of the skin. Eight days after the appearance of these signs, the two cows died. An ELISA test performed on both animals confirmed the presence of specific antibodies against *Besnoitia besnoiti*. A few weeks later, a 14-month-old bull showed the same clinical signs, such as fever and edema of the limbs and scrotum. However, the ELISA test was negative one week after the onset of clinical signs. After two weeks, although the clinical signs were lighter, the test was repeated and the result was positive. Subsequently, the bull was slaughtered even though it was not showing several clinical signs.

### 2.2. Farm B

#### 2.2.1. Serological Findings

On this farm, a serological survey was conducted on a total of 72 heads (69 female, 3 male), and 57 animals tested positive for specific antibodies (79.17%) by ELISA (56 female, 1 male). Those that were positive belonged to a highly variable age group ranging from 1 month to 13 years (Table 2).

#### 2.2.2. Clinical Signs

In July, two Limousine bulls were introduced to the farm from a farm about 7 km away. In September, one of the two bulls, 14 months old, had a high fever and, after one week of prolonged hyperthermia that exceeded 41 °C, died. In that period, there was no suspicion of *B. besnoiti* infection, even though a reduction in fertility was observed, and no further tests were performed or organs collected.

**Figure 1:**
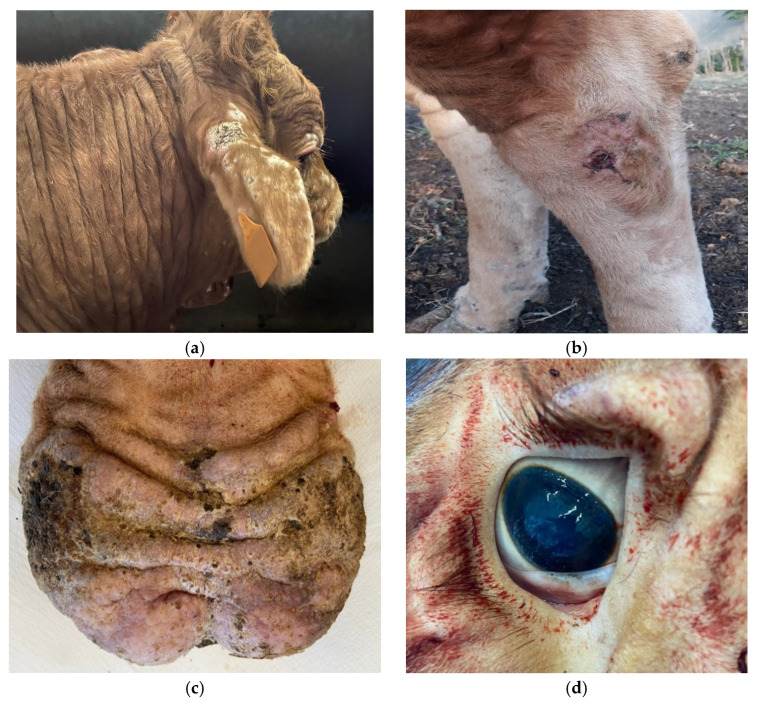
Chronic skin lesions in the 15-month-old bull of farm B: (**a**) alopecia, scabs, and scleroderma in ear, snout, and periocular region; (**b**) edema and eschar of the hind limbs; (**c**) edema and hyperkeratosis of the scrotum; (**d**) ocular–conjunctival mucous membrane cyanosis.

Later, the other bull, 15 months old, showed mild clinical signs related to the chronic phase of besnoitiosis, such as fever and debilitation. About two months later, a clinical examination of the bull reported fever for a whole week, with temperature rising to 41.5–42 °C. A few days later, the bull presented a scrotal swelling and subsequent increasing in testicular volume, thickening of the skin above the nose, edema of joints (anterior and posterior limbs), and conjunctival hyperemia with lacrimation. About two weeks after the onset of clinical signs, furfuraceous skin lesions appeared, which led to generalized scleroderma. Skin thickening was mostly located in the ears, periocular region, nasal planum and muzzle, dewlap, and limbs; furthermore, alopecia and scabs were also observed on some other areas (Figure 1a,b). The scrotum, initially edematous, showed areas of marked hyperkeratosis, sometimes with the presence of eschars (Figure 1c), but the lesions were not itchy or painful to the touch. In addition, the bull showed dehydration, cyanosis of the buccal and ocular–conjunctival mucous membranes (Figure 1d), and a generalized increase in explorable lymph node volume. The bull was also tachycardic and dyspneic, but the main vital functions were still maintained. Since the onset of fever, the antibiotic (oxytetracycline hydrochloride, 3 mg/kg bodyweight, injected intramuscularly every day for five consecutive days) and anti-inflammatory (ketoprofen, 3 mg/kg body weight for three days) treatments did not have any positive effects and the progression of the disease led to a marked worsening of the general conditions as well as the appearance of skin alterations.

Considering the positive reaction to the specific antibody of *Besnoitia besnoiti* detected on the serum by ELISA and the general suffering of the animal, the bull was slaughtered. Histological findings confirmed the presence of the parasite.

#### 2.2.3. Histological Findings

**Figure 2:**
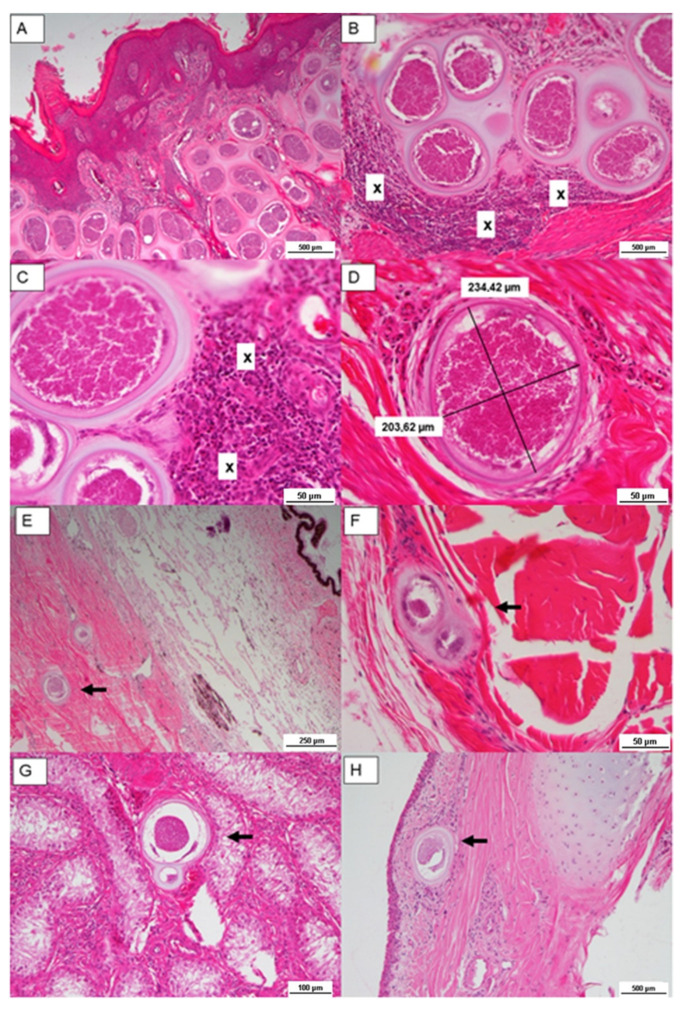
Histological section. Multiple *Besnoitia* spp. cysts (**A**–**D**) in dermis with parasitophorous vacuole lined by a capsule containing thousands of banana-shaped zoites. Note: x denotes monocytic infiltrates; (**E**) tissue cyst in eye; (**F**) in tendon; (**G**) in testicle; (**H**) in nictitating membrane.

In order to confirm the infection, samples of the skin lesion, eyes, tendons, and testicles were taken from the 15-month-old bull of farm B. Histological examination confirmed the suspicion of infection; in particular, Besnoitia tissue cysts containing bradyzoites were present in all skin specimens, in the dermis, in the panniculus adipose, in the underlying muscle layer, in tendons, in the eyes (Figure 2E,F), and in the tongue and testicles (Figure 2G,H).

The skin samples were mainly affected and frequently associated with epidermal hyperplasia. The superficial dermis showed many parasites encysted in connective tissue. The cysts, shaped as circular bodies, were 200–300 µm in diameter and comprised a thick, lamellated, eosinophilic double wall, which contained a parasitophorous vacuole with thousands of banana-shaped bradyzoites (Figure 2A–C). Some cysts were surrounded by moderate numbers of lymphocytes, macrophages, and eosinophils, and there was mixed nonsuppurative inflammation with rare multinucleated giant cells. Cyst walls showed distinctive features of *Besnoitia* spp. cysts (thick, double-walled), which allow it to be distinguished from other genera within the Sarcocystidae family (Figure 2D) [1,22]. Sometimes, the lysis of cysts caused an intense granulomatous reaction, with many multinucleated giant cells surrounding the capsular wall.

## 3. Discussion

In the last two decades, bovine besnoitiosis has been recognized as an endemic pathogen in many European countries, including Italy, where it is considered an emergent disease since autochthonous outbreaks were reported mostly in northern and central areas [23,24,25] and rarely in southern regions [21]. Certainly, the growing attention to the veterinary risk may ensure efforts to limit the spread of infection, by mapping the outbreaks and allowing local veterinary authorities to eventually establish adequate measures of control and surveillance. In Sicily, there is a lack of information about this disease: firstly, because the clinical signs often assigned to other infections; secondly, because it is an emergent disease. Indeed, the first report in Sicily is dated June 2016 [20]. This is related to the difficulty of making an early diagnosis, due to the clinical signs of the acute phase of besnoitiosis not being specific. On the contrary, the clinical signs of the chronic phase may be mistaken for other bovine skin diseases like scab (sarcoptic or psoroptic), dermatophytosis, dermatopathies due to deficiency disease, or chronic toxic syndromes [11,14,23,24,26,27].

This study confirms the presence of autochthonous outbreaks of bovine besnoitiosis in Sicily, a confirmation that is strengthened by the high seroprevalence within the two farms (61.6% of animals showed anti-*B. besnoiti* antibodies), related to the low prevalence of clinical signs in seropositive animals. The ELISA test played an important role in this study because it was a robust tool for rapid diagnostic screening in a large number of animals, even though the diagnostic period from infection to seroconversion is still an unresolved issue [16]. However, for detection of IgM and avidity, ELISA improved early in vivo diagnosis. In fact, when the IgM are positive but the IgG are negative, it is indicative of an acute infection, whereas when the IgG are positive and are accompanied by low avidity values, it confirms a recent infection [28]. Above all, it was useful to discriminate the chronic stage from the acute stage in animals recently introduced to the herd, such as the 14-month-old bull of farm A, which tested negative at the beginning of infection and positive one week after the appearance of clinical signs.

Considering the two farms involved in this study, it was possible to distinguish two different epidemiological situations: for farm A, the seroprevalence was 28.20%, while for farm B, it was 79.17%, which is significantly higher than in studies reported from central [12] and southern Italy [25]. Nevertheless, for both farms the percentage of clinical cases was lower than 2% (1.17% on farm A; 1.44% on farm B). The few acute clinical cases were probably linked to the high percentage of seropositive animals that showed clinical signs compatible with the chronic phase. These animals were born on the farms and not imported from endemic regions. Therefore, when new animals were introduced to the herd, they became infected and showed clinical signs of infection related to the acute phase, usually after an incubation period of 6–10 days [11], as in the case of the two bulls from farm B. These bulls were in contact with a positive herd of infected heads (around 80% according to the ELISA results). The bulls probably became infected through the following: mechanical transmission by blood-sucking arthropods, such as biting flies or tabanids [5,26]; less commonly, through the ingestion of oocyst spread in the environment from a definitive host (still unknown) [29,30]; or natural mating, since parasitic cysts lie in superficial mucosal tissues in the vagina and in the penis [31]. However, it cannot be discounted that the bulls were infected by iatrogenic means through hypodermic needles [5], probably through the use of the same gun-injector to treat the animals with tuberculin. The second 15-month-old bull on farm B showed typical disease progression, displaying clinical signs of the acute and chronic stages of besnoitiosis, without the risk of another skin infection. Histopathological investigations confirmed the diagnosis by identifying the presence of banana-shaped bradyzoites (typical for this protozoa) in tissue cysts [26]. Interestingly, there were seropositive animals ranging from one month to 13 years of age, different to data reported by Gutierrez et al. [32], in which, however, the number of tested animals was much higher than this study and age was considered as a risk factor.

This study confirms that bovine besnoitiosis is endemic in this area, considering the very low number of clinical cases in spite of the high seroprevalence [33]. However, further studies on transmission and epidemiology are necessary in this area.

## 4. Materials and Methods

In this study, two cattle farms 5 km apart in northwestern Sicily were analyzed. On these farms, Limousine cows were kept to produce meat in free-stall mode, with free access to pasture; no artificial insemination was carried out on the farms.

All animals were evaluated via an accurate analysis of remote and recent history, particularly focused on the new stock. The herd data showed that all animals were autochthonous because they were born on the farms or bought from local breeders. There were no animals imported from abroad. Moreover, the clinical status was evaluated in both herds, monitoring either general body conditions or peculiar clinical signs of besnoitiosis. No clinical inspection was carried out for cysts in eyes and the vulval vestibule.

### 4.1. Characterization of Farms

#### 4.1.1. Farm A

This farm is located in Palermo (37′61′163° N; 14′08354° E) and there were 39 Limousine cattle, 30 females and 9 males. The clinical history of this farm reported two cows and one bull showing clinical signs of besnoitiosis in April 2020. After a suspicion of infection, the herd was tested by ELISA in July. Samples of serum were collected from all 39 animals.

#### 4.1.2. Farm B

This farm is located in Palermo (37′68′072° N; 14′14733° E) and there were 72 cattle Limousine, 69 females and 3 males. On this farm two bulls, Limousine, were introduced in July 2020, one 14 months old and one 15 months old, from a nearby farm that had never reported any cases of besnoitiosis. In September 2020, the 14-month-old bull died after showing clinical signs potentially related to besnoitiosis; unfortunately, the difficulty of delivering an early diagnosis discouraged the veterinarian from collecting serum and organ samples. After a few weeks, the second bull, 15 months old, showed typical signs of besnoitiosis. Thus, it was slaughtered and biopsy samples of different tissues were collected for histological examination. In October 2020, the whole herd was tested by ELISA. Serum samples were collected from all 72 animals.

### 4.2. ELISA

Blood samples taken from the tail vein of cattle were centrifuged at 3500 rpm for 5 min to collect the serum fraction. A commercial ELISA kit was utilized to detect specific antibodies in order to determine the seroprevalence in the herd. Enzyme-Linked Immunosorbent Assay (ELISA) was performed on a total of 111 serum samples using the diagnostic kit “ID Screen^®^ Besnoitia Indirect” (IDvet, Grabels, France). This kit detects the specific antibodies in bovine serum or plasma and is based on the ELISA indirect method using a *Besnoitia besnoiti*-purified antigen extract [34]. The components of this ELISA commercial test are a coated antigen (*Besnoitia besnoiti*-purified antigen) and a conjugate (antiruminant IgG-HRP conjugate-concentrated 10×). Basically, the even-numbered plate columns are coated with a *Besnoitia besnoiti*-purified antigen, and the samples together with controls are added to the even- and odd-numbered plate columns to form an antigen–antibody complex when specific antibodies are present. Subsequently, an antiruminant peroxidase (HRP) conjugate is added to fix the complex with conjugate. Lastly, after washing, the substrate solution and the stop solution are added. The complex reacts and each well turns a specific color depending on the quantity of specific antibodies: yellow in the presence of antibodies; transparent in the absence of antibodies. The microplate is read at 450 nm. Results are calculated with respect to the corrected sample OD (optical density) obtained from each well by spectrophotometer and calculated by specific IDvet software to obtain the S/P ratio (Equation (1)):

(1) $$S/P=\frac{OD\;even\;well-OD\;odd\;well}{OD\;\;even\;well\;\left(positive\;control\right)\;-\;OD\;\;odd\;well\left(positive\;control\right)}*100.$$

The samples are considered negative if presenting a S/P value less than or equal to 25, doubtful if less than 30 and greater than 25, and positive if greater than 30.

### 4.3. Histological Examination

After the slaughtering of the 15-month-old bull of farm B, samples from the head, testicles, limbs, and tendons (deep digital flexor and superficial digital flexor) were collected for histopathological investigation. Different fragments were taken from observed lesions using both incisional and punch biopsy (Biopsy Punches 5 Ø, KAI Industries Co., Tokyo, Japan). Samples obtained from the skin, muscle, tendons, eyes, appendages, tongue, and testicles were fixed in 4% paraformaldehyde until they were dehydrated, cleared, and infiltrated with paraffin in a tissue processor. Subsequently, 4-μm slices were obtained and placed on glass slides. The glass slides were dried overnight at 37 °C and dewaxed by xylene for 20 min. The slides were treated with a descending alcohol series (100%, 95%, 75%, and 50%) and washed in distilled water. Subsequently, they were stained with hematoxylin and eosin (HE) and, after the ascending scale of alcohols (50%, 75%, 95%, and 100%), they were clarified in xylene. Lastly, the slides were mounted in an acrylic mounting medium (Eukitt^®^, O. Kindler GmbH, Freiburg, Germany) and, through a Leica DMLB microscope connected with a Nikon camera, a morphology analysis was performed [35].

## 5. Conclusions

The aim of this study was to report on a new case of autochthonous outbreak of *B. besnoiti* in Sicily with high seroprevalence for two nearby farms. Considering the target population, it could be claimed that this disease affected more than half of the herd and not a specific age class, since even calves were found to be seropositive. The low prevalence of clinical cases in relation to the high seroprevalence could mean that bovine besnoitiosis is endemic in this area.

The clinical behavior of the outbreak allowed us to differentiate the stages and progression of the disease, whereas histopathology clearly confirmed the presence of parasites in all the superficial cysts and in deeper tissues.

Finally, considering the severe lesions and possible economic losses related to bovine besnoitiosis, this study also aimed to increase knowledge about this disease and confirm its presence in Sicily. Moreover, with regard to other reports from Italy, further studies on transmission and epidemiology are necessary to investigate the prevalence and incidence of bovine besnoitiosis, in order to eventually implement specific surveillance and/or a control program to prevent the spread of the disease.

## Figures and Tables

**Table 1:** Distribution by category and age class of *Besnoitia besnoiti* positive heads on farm A (ELISA).

Category	Age Class	Number of Positive Animals	Antibodies Values (S/P) ^1^
Calves (male)	<12 months	1	53.32
Females	1–4 years	4	94.08
Females	4–6 years	3	193.71
Adult females	7–11 years	3	115.04

^1^ S/P ≤ 30 NEGATIVE; S/P ≥ 30 POSITIVE. This value is calculated by specific IDvet software based on optical density (OD).

**Table 2:** Distribution by category and age class of *Besnoitia besnoiti* positive heads on farm B (ELISA).

Category	Age Class	Number of Positive Animals	Antibodies Values (S/P) ^1^
Calves (males)	<12 months	3	59.74
Females and males	1–2 years	22 and 1	104.72 and 200.09
Females	3–6 years	10	105.03
Adult females	7–13 years	21	124.11

^1^ S/P ≤ 30 NEGATIVE; S/P ≥ 30 POSITIVE. This value is calculated by specific IDvet software based on optical density (OD).

## Data Availability

Not applicable.
